# US–Indian Relations: Formation of an Alliance or a Temporary Partnership?

**DOI:** 10.1134/S1019331622100069

**Published:** 2022-09-22

**Authors:** A. A. Davydov, A. V. Kupriyanov

**Affiliations:** Primakov National Research Institute of the World Economy and International Relations, Russian Academy of Sciences, Moscow, Russia

**Keywords:** India, United States, Joseph Biden, Donald Trump, Barack Obama, US foreign policy, US spatial policy, political space, Indian foreign policy

## Abstract

This article analyzes bilateral relations between India and the United States in the context of a new round of confrontational bipolarity between Washington and Beijing. The analysis of the historical dynamics of relations between the United States and India demonstrate that the US policy towards New Delhi has always been of an opportunistic nature and depended primarily on the events in southern Asia and the Indian Ocean, and on the dynamics of US relations with key Asian powers—the Soviet Union and China. India has never had an independent value for the United States. The existence of common values has always been used by both parties only to justify the next rapprochement between them and has always been determined by purely pragmatic considerations. At the same time, maintaining close relations with the United States is a strategic necessity for India, since the development of the Indian economy and the ability of New Delhi to balance between great powers depend on them. The authors come to the conclusion that today the imperatives of Washington and New Delhi have not changed significantly; therefore, there is no need to talk about a deep transformation of American–Indian relations.

## INTRODUCTION

The beginning of the third decade of the 21st century can be characterized as the period of the greatest intensification of international competition since the end of the Cold War. The strategic line of the largest states and international political associations towards strengthening political independence, observed in recent decades, is increasingly contributing to the diversification of their international relations. In parallel with this process, a reverse trend has recently been observed: throughout the 2010s, as US–Chinese tensions escalate, various states are seeking to fit into the logic of a new confrontational bipolarity in order to secure themselves and, with luck, benefit from the clash of giants.

One of the most striking examples of the intersection of these two processes was the accelerated strengthening of relations between the United States and India in the second half of the 2010s. Today’s mutual rhetoric, their foreign policy goal-setting, and bilateral ties are in stark contrast to the situation that existed twenty years ago, when relations could have been characterized as neutral–friendly at best.

The growth of the mutual strategic importance of these countries in the current conditions of the transforming structure of international relations raises a number of important research questions. First of all, they relate to the assessment of the qualitative state of bilateral relations between the United States and India. Are there any prerequisites for the long-term consolidation of these relations as allied ones, or are the dynamics observed today in the long term a market fluctuation under the influence of the international situation in the Asia–Pacific region? Does the structure of the bilateral relations being built allow them to be characterized as asymmetric, or are countries so far apart that they determine the line of strategic behavior completely independently, while maintaining equal subjectivity in international affairs?

US–Indian relations have traditionally been the focus of attention of both domestic and foreign researchers. Soviet and Russian Americanists studied them in the context of US foreign policy in southern Asia, regional conflicts, and the struggle against national liberation movements. Among such studies, one can separately note the class papers of V.A. Kremenyuk (1979; 1985), studies by N.S. Beglova (1984), A.D. Portnyagin (1977, pp. 189–203), and V.I. Batyuk (2021). This topic has also been studied by domestic Indologists: among the key fundamental papers, one can single out the monograph and numerous articles by S.I. Lunev (1987, 2018, 2020), as well as the classic general work and articles by F.N. Yurlov (2010, 2013). Individual aspects of the interaction between India and the United States were studied by N.B. Lebedeva (2019), A.I. Zakharov (2016), and E.P. Shavlai (2020).

Great attention is paid to the topic of bilateral relations by English-speaking, primarily Indian, researchers, many of whom work closely with American universities and think tanks. Relatively recent papers include monographs by R. Chaudhuri (1947), A. Bhardwaj (2018), N. Acharya (2016), a collective paper edited by S. Ganguly, E. Scobell, and B. Shoup (2006), as well as the paper by H. Pant and Y. Joshi (2015).

## THE UNITED STATES FOR INDIA, INDIA FOR THE UNITED STATES

The formation of US–Indian relations took place mainly under the influence of external factors and in relatively difficult circumstances. Back in the late 1930s, before India gained independence, the subject of the suffering of the Indian people under British rule was a concern in American intellectual circles, primarily due to historically widespread anti-British sentiment among them. Back in 1941, US President F.D. Roosevelt raised the question of the need for decolonization, which met Washington’s strategic interests in strengthening its economic position in new markets, before the Prime Minister of the United Kingdom, W. Churchill. Britain invariably rejected any attempts to influence the British position on India under the pretext that the Indian question was an internal affair of the empire, although the president repeatedly returned to this problem (Dulles and Ridinger, 2015; Rubin, 2011). For ordinary Americans, however, a direct acquaintance with Indian realities occurred during the Second World War, in which India became the base for operations of the American air and ground forces that acted against Japan in the Chinese and Burmese theaters of military operations (Raghavan, 2018). Accordingly, beyond the sympathies of the political class (initially determined by external reasons) and the interests of business, as well as the historically brief direct contact of citizens during the war, the United States had no other significant internal imperatives for developing relations with India.

India turned out to be a rather difficult partner in foreign policy as well. Its first steps in the foreign policy field demonstrated to Washington that India, although it supports the very idea of decolonization, is not going to change one master for another. Indian elites, who were in awe of their newly gained independence, were initially wary of communist expansion in southern Asia, trying to balance between the United Kingdom and the United States. Subsequently, the government of J. Nehru increasingly shifted towards neutrality, seeing India as the leader of a bloc of Asian and African post-colonial states (Singh, 1976, p. 46).

In Washington, the strategy for southern Asia was initially developed based on the need to contain the growth of the influence of communist forces supported by the Soviet Union, and subsequently by China.^1^ The bloc approach in foreign policy determined the State Department’s misunderstanding of the entire complexity of the situation that was developing in South and Southeast Asia, and led to unsuccessful steps: for example, in December 1947, the United States tried to put pressure on New Delhi, forcing Nehru to “immediately join the democratic camp,” which caused resentment among the Indian political elites (McMahon, 1994, p. 40). The visit of Prime Minister J. Nehru to the United States in 1949 helped resolve a number of misunderstandings, but did not lead to a breakthrough in relations and was characterized by both sides as a failure.

Over the ensuing decades, US–Indian relations developed in an uneven sine wave, where occasional ups were followed by deep downs. American politicians interested in strengthening the Western camp in the Cold War often perceived the Indian strategy as hypocritical and duplicitous. Thus, New Delhi was one of the first to recognize the People’s Republic of China in 1949, and in the Korean War of 1950–1953, India supported UN forces by sending a mobile hospital to Korea. The White House tried to strengthen its influence on Indian politics by sending cash and food aid. Between 1954 and 1971, Washington allocated $57 billion to New Delhi, of which $25 billion accounted for food aid (data in 2019 prices of the US Agency for International Development^2^). Thus, the United States not only contributed to solving the problem of hunger and poverty in India, but also provided support to its farmers.

At the same time, the United States actively strengthened cooperation with India’s key regional adversary, Pakistan, where elites were more sympathetic to Washington’s bloc policy. Islamabad joined the Southeast Asia Treaty Organization (SEATO) in 1954, and the Central Treaty Organization (CENTO) in 1955, which contributed to the buildup of American military assistance to Pakistan. For the period of 1954–1971, Pakistan received $5 billion worth of military aid and $1.6 billion worth of weapons, which exceeded the corresponding deliveries to India by five and three times respectively (AID data in 2019 prices, SIPRI data in current prices^3^). Despite assurances from Washington that it would not allow these weapons to be used against India, such a disproportion aroused concern in New Delhi.

A sharp thaw in relations between the United States and India occurred in the last years of D. Eisenhower’s rule and under J. Kennedy. They saw India as a strategic partner and key player in South Asia to deter the expansion of communist China. After the outbreak of the Indo–Chinese war in 1962, the United States provided India with significant assistance in weapons, ammunition, and military equipment, and after the defeat of India, helped restore the combat effectiveness of the Indian army. The issue of possible US intervention in the event of a new Sino-Indian conflict was seriously discussed, up to the use of nuclear weapons to protect India.^4^

By supporting India, Washington pursued its own goals and reluctantly made reciprocal concessions. By inciting India to move away from the principle of neutrality in order to turn it into an anti-Chinese foothold, the United States simultaneously tried to maintain good relations with both India and Pakistan, but gradually increased pressure on New Delhi, trying to induce it to make concessions on the Kashmir issue. Such a policy turned out to be erroneous and only led to an aggravation of bilateral contradictions, resulting in a new Indo–Pakistani war, which was only stopped by the joint efforts of the United States and the Soviet Union.^5^

The failure of the “carrot and stick” policy, the uncertain outcome of the war, and the aggravation of the situation in Vietnam significantly weakened the US position in the subcontinent and forced a qualitative review of its strategy in Asia and approaches to India. As Soviet–Indian cooperation strengthened, L. Johnson and R. Nixon, who replaced him, began rapprochement with the PRC in opposition to the Soviet Union and gradually moved away from supporting India, relying on Pakistan, an ally of China. This trend was clearly manifested during the third Indo–Pakistani war in 1971, when the United States accused India of aggression and sent the aircraft carrier Enterprise to the Bay of Bengal.

The crushing defeat of Pakistan in the war and the general reorientation towards strengthening ties with China contributed to a decline of the interest of the American elites in South Asia. The catastrophic outcome of the Vietnam campaign, Pakistan’s withdrawal from CENTO, and the revolutions in Iran and Afghanistan pushed South Asia to the periphery of the Cold War in the minds of American strategists. Despite the fact that the consistent position of New Delhi as the leader of the nonaligned movement was objectively more beneficial to the Soviet Union, the United States no longer tried to win India over to its side, confining itself to the gradual development of trade and economic ties. The prevailing set of circumstances, in fact, brought New Delhi out of the logic of bipolar confrontation.

The end of the Cold War seemed to create fertile ground for a qualitative change in the nature of US–Indian relations. However, the US strategic line towards India remained the same. The Clinton administration tried to force India to carry out structural reforms, demanded economic liberalization, repeatedly criticized New Delhi for violating human rights, and questioned the legitimacy of the actions of the Indian authorities in Kashmir (Zakharov, 2016). An additional problem in bilateral relations was the development of the Indian nuclear program, because of which the White House imposed sanctions on India in 1998 (Tellis, 2006).

A breakthrough in relations was achieved only after the United States, faced with a new threat, reconsidered its priorities in the Indian direction. The military campaign in Afghanistan that followed the terrorist attacks of September 11, 2001, significantly updated for Washington the importance of India’s geostrategic position and its experience in fighting Islamist separatists. In 2004, a “strategic partnership” between New Delhi and Washington was announced, which was based on common values and interests.^6^ An additional and very significant catalyst for the further strengthening of the US–Indian partnership was the growth of tension in relations between Washington and Beijing after the attempts of the Obama administration to increase cooperation with China within the framework of the so-called G2 failed.^7^ It is significant that the United States chose the Indian concept of the Indo–Pacific region, formulated back in 2007, as the conceptual geopolitical basis for the formation of a system to deter China.

Thus, the mutual strategic importance of the United States and India was determined primarily by the transformation of the global strategy of the United States. Washington’s policy towards New Delhi has always been opportunistic in nature and depended primarily on developments in neighboring countries and the region, as well as on US relations with the Soviet Union and China. When relations between Washington and Beijing improved, the need for India as a counterbalance to Chinese influence disappeared, and American political elites lost interest in it. The factor of common democratic values was used only to justify another attempt at Indian–American rapprochement. India, for its part, throughout the history of bilateral relations, proceeded from the fact that it needs US assistance to ensure security in the face of the Chinese threat, as well as to develop its economy. At the same time, the preservation of strategic autonomy remains an unconditional priority for New Delhi, and India does not perceive the Chinese threat as existential due to centuries of being in the same neighborhood.

## MODERN STRATEGY AND BILATERAL RELATIONS

The arrival of the D. Trump administration to the White House marked the consolidation of the conceptual vision of the US policy towards India that had been formed under B. Obama. The American Indo-Pacific strategy announced in November 2017 implied a qualitatively different positioning of India as a key US ally in South Asia and the Asia–Pacific Region.

From a strategic point of view, India has taken a central place in the implementation of the US Indo-Pacific strategy in the Asia–Pacific Region and South Asia. The Trump administration justified the need for this strategy at a conceptual level to strengthen the principles of freedom and openness in regional relations in order to counteract the aspirations of the revisionist countries, primarily China. Most likely, this line will be pursued further, given the existence of a cross-party consensus on this issue in Congress.

**At the macro level of the Asia–Pacific Region and Southeast Asia,** the United States, without hiding the anti-Chinese orientation of its actions, is trying to build new or strengthen existing multilateral formats of cooperation between the countries of the region, emphasizing the central importance of India in them. At the moment, the political interests of the United States and India in the field of security largely coincide. Both countries are interested in containing Beijing, weakening its foreign policy positions, and disrupting the Belt and Road project. Thus, a significant step was the resuscitation in 2017 and the rise to the ministerial level in 2019 of the Quadrilateral Security Dialogue (Quad) between the United States, Japan, India, and Australia, which addresses the issues of naval security, cybersecurity, working against terrorism, and infrastructural interconnectedness (Mishin, 2020). The United States points to the open nature of the dialogue and the prospect of its expansion. In addition, following the logic of supporting multilateral formats, Washington emphasizes the role of ASEAN as a key forum for the development of regional economic relations and actively supports the admission of India to APEC.^8^

Joint exercises of the United States and India, as well as their potential allies, have intensified. Thus, in 2020, New Delhi invited Australia to participate in the Malabar trilateral naval exercises, which India had not done before in order to avoid accusations of anti-Chinese maneuvers.^9^

Meanwhile, in the long run, the views of Washington and New Delhi diverge significantly. If the United States is interested in maintaining world hegemony, or at least forming an alliance powerful enough to prevent Beijing from dominating, then India’s goal is to achieve recognition from China as a great power and to create a separate center of power and a zone of influence around it, which should include all of South Asia (except Pakistan) and the Indian Ocean (Brewster, 2014, pp. 35–37). Many representatives of Indian political and expert circles are pro-American, arguing that there is no alternative to further rapprochement with the United States. However, the ruling alliance, led by the Bharatiya Janata Party, advocates a multi-alignment strategy in which India would develop ties with all major players and their alliances while maintaining strategic autonomy (Jaishankar 2020).

**At the level of the South Asian region,** the United States is using India as a springboard to put pressure on China. Thus, with the accession of D. Trump to the White House, the United States intensified the practice of supporting Tibetan separatism that had been started under George W. Bush, Jr., seeking to attract India and Nepal to it. Since 2018, Washington has been allocating at least $8 million annually to non-governmental organizations (NGOs) in the Tibet Autonomous Region of China. NGOs in India and Nepal receive upwards of $6 million each year for programs for the preservation of the Tibetan cultural heritage, educational projects, and the education of a new galaxy of Tibetan leaders. Furthermore, the United States provides over $3 million annually to strengthen the work of Tibetan state institutions^10^.

With the next round of aggravation of the Indo-Pakistani conflict in 2016, the United States chose not to intervene in the situation, fearing that India’s unilateral support would significantly damage relations with Pakistan, which could seriously aggravate the situation in Afghanistan. In connection with this fear, the Trump administration decided not to impose sanctions against Pakistan and not to designate it as a state sponsor of terrorism and not to deprive it of its status as a Major non-NATO ally (all these measures were seriously considered at the beginning of Trump’s presidency).^11^ After the repeal in August 2019 of Article 370 of the Indian Constitution, which guaranteed autonomy to the state of Jammu and Kashmir, Washington refrained from assessing this move, instead offering the services of an intermediary in negotiations between New Delhi and Islamabad.^12^

At the same time, the US made certain concessions to India so as not to spoil relations with a key partner in the framework of the Indo-Pacific strategy. Washington de facto recognized the change in the status quo in Kashmir and did not impose (at least for now) sanctions against India for the purchase of Russian weapons (primarily the S-400) and for maintaining a minimum share of oil imports from Iran (in 2018, India imported $13.3 billion worth of oil from Iran, which accounted for 7.9% of all Indian oil imports, and in 2019 the volume decreased by 78.2% to 2.9 billion dollars, or to 1.9% of oil imports).^13^ Instead of putting pressure on New Delhi, Washington is seeking to offer an alternative whenever possible, for example, India, the United States, and Israel began to build trilateral cooperation between companies from Silicon Valley, Tel Aviv, and Bangalore to develop their own 5G technologies with the potential to involve other US allies in this cooperation.^14^

**At the bilateral level,** the United States is taking steps to develop a strategic military partnership with India. Qualitative shifts have taken place within the framework of the US–Indian Defense Technology Trade Initiative, launched in 2012.^15^ During the presidency of D. Trump, the United States entered into agreements with India to simplify the creation of joint production chains in the field of the military-industrial complex and to exchange information in the field of security, which should increase the compatibility of various types of weapons of the United States and India. An agreement on the exchange of satellite and topographic data for long-range navigation is under development.

The United States has moved India into the category of states with the most simplified export control regime for security-sensitive goods, services, and technologies (it includes 37 countries). This regime applies to 26 of the 30 NATO countries (except for the United States itself, Albania, Montenegro, and North Macedonia), five of the 18 main allies outside NATO and six countries that do not formally have allied relations with the United States (Austria, Finland, India, Ireland, Sweden, and Switzerland).^16^

The United States is increasing arms exports to India: the US now accounts for 0.64 of 2.9 billion dollars, or 21.6% of India’s total arms imports. According to this indicator, the United States reached the third place after Russia (1.1 billion dollars or 39.5%) and Israel (0.7 billion dollars or 24.7%). It is important to note that Russia’s presence in the Indian arms market has noticeably declined over the past six years, both in absolute terms (from 3.8 billion dollars in 2013) and in share.^17^

Washington is also purposefully developing trade and economic cooperation with New Delhi. Although the proposal made by D. Trump in 2018 to create a free trade zone with India was never implemented, the United States greatly increased its direct investment in India, reaching reached $43.6 billion, $13.8 billion dollars of which was invested in 2020.^18^ Total trade turnover increased from $59.5 billion in 2009 to $146.1 billion in 2019, thus, trade is carried out with a growing deficit in favor of India (from $8.3 billion to $28.8 billion).^19^ This trend eventually forced the Trump administration to take tough measures: it abolished the preferential trade regime with India, accusing it of promoting protectionism and abusing the status of a country with a developing economy. Nevertheless, the measures that the United States applied against India turned out to be much softer than those applied to China.^20^

For India, bilateral trade and economic relations are much more important than for the United States. The United States is one of India’s largest trading partners along with China: they account for 11.8% of all Indian foreign trade, while India’s share is only 2.1% of the US foreign trade (10th place). Similarly, with investments: if India accounts for only 2% of FDI in the United States ($5 billion out of $246 billion^21^), then the United States is 8% of FDI in India, and if one considers American allies, then their total share exceeds 30%. Thus, for India, trade with the United States and the influx of American investment play a critical role, and breaking these ties will entail serious economic difficulties, but for the United States, India is a relatively insignificant trading partner that can be sacrificed if necessary.

Finally, US–Indian relations are influenced by the mood of the Indian community in the United States, which in 2018 numbered 4.1 million people (2% of the total US population). Today, the Indian community in the United States holds a steady lead in terms of income per person among all diasporas in the country, since it mainly consists of highly qualified specialists (Thomas, 2018). Nevertheless, the high integration of the diaspora into business and political circles has not led to an increase in pro-Indian lobbying. For example, participation in the election for the post of US Vice President K. Harris did not mobilize the diaspora, as happened with B. Obama and African Americans in 2008. On the contrary, sympathy for the Republicans is growing in the Indian community due to the fact that the Democrats, trying to win Muslim votes, have repeatedly criticized the Indian authorities for violating human rights in Kashmir and reproached the Republican administration for ignoring this problem. As a result, some Indians have abandoned their traditional support for the Democratic Party,^22^ despite the initiatives of the Republicans to restrict immigration, which is painful for the diasporas.^23^

The recent steps of the Biden administration towards India (in particular, the warm welcome extended to N. Modi during his visit to the United States in September 2021, and the fact that during his visit the American side avoided raising the subject of human rights violations) instigated tough criticism from American human rights activists and part of the Democratic Party activists. However, the new US leadership has made it clear that it plans to further develop ties with New Delhi, seeing it as a counterbalance to Beijing, and is not ready to criticize the Modi government openly for violating human rights. According to high-ranking American diplomats, the geostrategic importance of India for the United States is so great that it is not advisable to spoil relations with it at the current stage.^24^

## CONCLUSIONS AND PROSPECTS

The dynamics of the development of US–Indian relations clearly demonstrates their dependence on the transformation of the system of international relations in general and the global strategic goal-setting of the United States in particular. While New Delhi has always been more interested in the development of bilateral trade and economic and investment ties, Washington saw India primarily as a regional power, the potential of which could be used to curb communist, and then just Chinese, expansion, as well as to fight against Islamist extremism and terrorism.

The structure of bilateral relations is largely asymmetric. This feature, however, does not give grounds to talk about the complete dependence of India on the United States. The latter is stepping up its military–political cooperation with New Delhi in the area of trade in weapons and defense technologies, trying to include India in multilateral anti-Chinese coalitions. However, India advocates a more inclusive approach to cooperation within its Indo-Pacific “vision” and does not view China as an existential enemy and Russia as an adversary. There is a powerful pro-American lobby in Indian political and expert circles that promotes the idea that further rapprochement with the United States has no alternative, but the Bharatiya Janata Party–led alliance in power prefers to maintain strategic autonomy. India’s aspirations look much more modest than the American ones and allow reaching an agreement with China on the division of spheres of influence, which could potentially bring India out of active confrontation with China.

The increased trade, economic, and military–technical cooperation between the countries did not lead to political steps by the United States towards India on sensitive issues. Moreover, Washington retains in its arsenal the ability to impose sanctions for the supply of Russian S-400s, for human rights issues, and for New Delhi’s policy in Kashmir. The declared strategic partnership is not accompanied by the US signing binding defensive treaties or transferring India to the status of a major ally outside NATO. In general, this line of behavior coincides with the American approach during the Eisenhower and Kennedy years, and also additionally emphasizes the situational nature of increased cooperation.

## Footnotes

1 Democratic Party Platform 1952, The American Presidency Project. https://www.presidency.ucsb.edu/documents/1952-democratic-party-platform. Cited August 6, 2021.

2 USAID, The complete Foreign Aid Explorer dataset, USAID. https://explorer.usaid.gov/prepared/us_foreign_aid_complete.csv.

3 SIPRI Arms Transfers Database, Importer/exporter TIV tables, SIPRI. http://armstrade.sipri.org/armstrade/page/values.php; USAID: Ibid.

4 JFK, aides considered nuclear arms in China–India clash, Taipei Times. http://www.taipeitimes.com/News/world/archives/ 2005/08/27/2003269368.

5 A. Braterskii, How the USSR reconciled India and Pakistan, Gazeta.ru, January 10, 2016. https://www.gazeta.ru/politics/ 2015/12/29_a_8002691.shtml.

6 United States–India Joint Statement on Next Steps in Strategic Partnership, The U.S. Department of State. https://2001-2009.state.gov/r/pa/prs/ps/2004/36290.htm.

7 The United States and China: A G-2 in the Making?, Brookings. https://www.brookings.edu/articles/the-united-states-and-china-a-g-2-in-the-making/.

8 Deputy Secretary Biegun Remarks at the U.S.–India Strategic Partnership Forum, The Department of State. https://www.state.gov/deputy-secretary-biegun-remarks-at-the-u-s-india-strategic-partnership-forum/.

9 India is set to invite Australia to join controversial naval exercise with the US and Japan to counter China’s aggression in the region after deadly border clash and COVID-19 origin row, Daily Mail. https://www.dailymail.co.uk/news/article-8543727/ India-calls-Australia-help-contain-threat-China-Malabar-naval-exercises.html.

10 Appropriations Status Table, The Congressional Research Service. https://crsreports.congress.gov/AppropriationsStatusTable/ Index/AppropriationsStatusTable?id=2021.

11 Remarks by President Trump on the Strategy in Afghanistan and South Asia, The U.S. Department of State. https://www.whitehouse.gov/briefings-statements/remarks-president-trump-strategy-afghanistan-south-asia/.

12 Remarks by President Trump in Press Conference, The White House. https://www.whitehouse.gov/briefings-statements/ remarks-president-trump-press-conference-4.

13 List of supplying markets for a product imported by India, Trademap. https://www.trademap.org/tradestat/Country_SelProductCountry_TS.aspx?nvpm=1%7c699%7c%7c%7c%7c27%7c%7c%7c2%7c1%7c1%7c1%7c2%7c1%7c2%7c1%7c1%7c1.

14 From T3, the India–US–Israel tech alliance can become T11, Observer Research Foundation. https://www.orfonline.org/ expert-speak/from-t3-the-india-us-israel-tech-alliance-can-become-t11-73161/.

15 U.S. and India Deepen Bilateral Defense Trade, Sign Two Defense Technology and Trade Initiative Agreements, The U.S. Department of Defense. https://www.defense.gov/Newsroom/ Releases/Release/Article/2044183/us-and-india-deepen-bilateral-defense-trade-sign-two-defense-technology-and-tra/.

16 BIS License Exception Statistics, The U.S. Department of Commerce. https://bis.doc.gov/index.php/documents/technology-evaluation/ote-data-portal/2173-sta-use-july-2011-december-2016/file.

17 SIPRI: Ibid.

18 Quarterly Fact Sheet on Foreign Direct Investment (FDI) from April 2000 to March 2021, Department for Promotion of Industry and Internal Trade. https://dpiit.gov.in/sites/default/files/FDI_Factsheet_March,21.pdf.

19 India, Office of the U.S, Trade Representative. https://ustr.gov/countries-regions/south-central-asia/india.

20 U.S.–India Trade Relation, The Congressional Research Service. https://fas.org/sgp/crs/row/IF10384.pdf.

21 United States: Foreign Investment, Santander Trade. https://santandertrade.com/en/portal/establish-overseas/united-states/foreign-investment.

22 Kashmir issue draws Indian–Americans to Republican camp, India Today. https://www.indiatoday.in/world/story/kashmir-issue-reasons-indian-american-shift-donald-trump-us-1722583-2020-09-17.

23 Why Trump’s H-1B visa freeze will hurt India most, BBC. https://www.bbc.com/news/world-asia-india-53150214.

24 N. Toosi, Biden needs India to counter China, but it comes with a cost, Politico, 2021. https://www.politico.com/news/ 2021/09/23/biden-modi-india-human-rights-china-514041.
